# Development of a Mucoadhesive and an in Situ Gelling Formulation Based on κ-Carrageenan for Application on Oral Mucosa and Esophagus Walls. II. Loading of a Bioactive Hydroalcoholic Extract

**DOI:** 10.3390/md17030153

**Published:** 2019-03-05

**Authors:** Barbara Vigani, Silvia Rossi, Matteo Gentile, Giuseppina Sandri, Maria Cristina Bonferoni, Valeria Cavalloro, Emanuela Martino, Simona Collina, Franca Ferrari

**Affiliations:** 1 Department of Drug Sciences, University of Pavia, Viale Taramelli, 12-27100 Pavia, Italy; barbara.vigani@unipv.it (B.V.); matteo.gentile01@universitadipavia.it (M.G.); giuseppina.sandri@unipv.it (G.S.); cbonferoni@unipv.it (M.C.B.); simona.collina@unipv.it (S.C.); franca.ferrari@unipv.it (F.F.); 2 Department of Earth and Environmental Science, University of Pavia, Via S. Epifanio, 14-27100 Pavia, Italy; valeria.cavalloro01@universitadipavia.it (V.C.); emanuela.martino@unipv.it (E.M.)

**Keywords:** oral mucositis, *Hibiscus sabdariffa* extract, κ-carrageenan, in situ gelation, mucoadhesion, antioxidant, anti-inflammatory properties

## Abstract

The aim of the present work was to load a *Hibiscus sabdariffa* (HS) hydroalcoholic extract into in situ gelling formulations for the treatment of oral mucositis and esophagitis. Such formulations, selected as the most promising options in a previous work of ours, were composed by κ-carrageenan (κ-CG), a sulfated marine polymer able to gelify in presence of saliva ions, hydroxypropyl cellulose (HPC), used as mucoadhesive agent, and CaCl_2_, salt able to enhance the interaction κ-CG/saliva ions. HS extract, which is rich in phytochemicals such as polyphenols, polysaccharides and organic acids, was selected due to its antioxidant and anti-inflammatory properties. For HS extraction, three different methodologies (maceration, Ultrasound Assisted Extraction (UAE) and Microwave Assisted Extraction (MAE)) were compared in terms of extraction yield and extract antioxidant activity, revealing that MAE was the best procedure. Rheological and mucoadhesive properties of HS-loaded formulations were investigated. Such formulations were characterized by a low viscosity at 25 °C, guaranteeing an easy administration, a proper in situ gelation behavior and marked elastic and mucoadhesive properties at 37 °C, functional to a protective action towards the damaged mucosa. Finally, the biocompatibility and the proliferative effect of HS-loaded formulations, as well as their antioxidant and anti-inflammatory properties, were proved in vitro on human dermal fibroblasts.

## 1. Introduction

Carrageenans (CGs) are an important class of hydrophilic pharmaceutical polymeric excipients obtained by extraction with water or aqueous alkali from some members of marine red seaweeds of the class Rhodophyceae, such as *Chondrus*, *Eucheuma*, *Gigartina* and *Hypnea*. CGs are sulfated polysaccharides, consisting of an alternating linear chain of galactose and 3,6-anydrogalactose, which can be classified according to the degree of substitution on their free hydroxyl groups. Depending on the number and position of the ester sulfate groups, three primary classes of CGs, named kappa (κ), iota (ι) and lambda (λ), are recognized. In particular, κ-CG, having one negative charge per disaccharide residue, is well known to obtain gels in presence of certain cations, such as K^+^, Na^+^, Mg^2+^ and Ca^2+^ [1,2,3].

In a previous work of ours [4], in situ gelling formulations based on κ-CG were developed for the treatment of oral mucositis and esophagitis. κ-CG was chosen for its ability to gelify in presence of saliva ions. The formulations also contained hydroxypropyl cellulose (HPC), employed as mucoadhesive agent, and CaCl_2_ that was proved to enhance, at a low concentration (0.04% w/w), the interaction between κ-CG and saliva ions. Different κ-CG, HPC and CaCl_2_ concentrations were investigated in order to obtain formulations able to interact with saliva ions, producing a gel capable of adhering to the damaged mucosa. The developed formulations were characterized by: (i) an easy administration, due to their low viscosity at room temperature, (ii) a protective action towards the mucosa, related to their marked elastic properties at 37 °C, and (iii) mucoadhesion properties.

These promising results prompted us to investigate the possibility of loading the previously developed formulations with a bioactive hydroalcoholic extract intended for the treatment of the oral mucositis and esophagitis.

*Hibiscus sabdariffa* Linn. (HS), also named roselle, red sorrel or karcadè, is an annual herbaceous subshrub belonging to the family of Malvaceae and is commonly distributed in tropical and subtropical regions [5,6,7]. The pharmacological properties of HS have been extensively studied for years, preparing extracts using several solvents (water, alcohols alone or in mixtures) and evaluating their biological effects [8,9,10,11,12,13,14,15]. All the HS extracts are characterized by the presence of phenols, polyphenols, anthocyanins and organic acids, such as citric, tartaric, malic, ascorbic acids and others, which are responsible for high antioxidant and anti-inflammatory properties [16,17,18,19]. These functions enhance the healing process by modulating the production of reactive oxygen species and pro-inflammatory cytokines that are responsible for the amplification of the damage [20]. In the present work, on the basis of our experience [21,22,23,24,25], we experimented different HS extraction methodologies (maceration, Ultrasound Assisted Extraction (UAE) and Microwave Assisted Extraction (MAE)), using a hydroalcoholic solvent. To obtain extracts rich in antioxidant metabolites, the mixture ethanol/water 80/20 v/v was used as extracting solvent in accordance with literature [26]. The different extracts were compared in terms of both extraction yield and antioxidant activity in order to select the most convenient approach.

Two formulations, indicated with the name BLANK 1 and BLANK 2, containing 0.04% w/w CaCl_2_, 1% w/w HPC and different κ-CG concentrations (0.6% and 0.4% w/w, respectively) were loaded with HS extract (0.2% w/w). Such formulations, after dilution to a 3: 1 weight ratio with artificial saliva or distilled water [4,27,28], were subjected to viscosity measurements at increasing shear rates in order to verify if the presence of the extract affects the formulation gelling capability. In addition to viscosity, the viscoelasticity of the vehicle could play an important role on its ability, once administered, to withstand the physiological removal action exerted by saliva and by the mechanical stress produced by the movement of the walls of the oral cavity [29]. For this reason, all formulations, loaded and not, have been subjected to dynamic oscillatory measurements at 37 °C after dilution in artificial saliva [30,31,32,33,34,35].

Thereafter, the mucoadhesive properties of all the formulations under investigation were studied: the samples were submitted to mucoadhesion measurements by means of a tensile test, using porcine gastric mucin as biological substrate [36,37].

In order to verify the biocompatibility of the developed formulations, the vehicle containing the highest concentration of κ-CG, unloaded and after loading with HS extract, was subjected to cytotoxicity and cell proliferation tests on human fibroblasts [38,39]. The developed formulations should be able to deliver HS bioactive substances without impairing their biological activity. Therefore, the capability of the formulations to protect cells from oxidative stress induced by hydrogen peroxide was also investigated in comparison with HS extract [40]. Moreover, the anti-inflammatory properties of the HS extract and HS-loaded formulations have been evaluated on fibroblasts inflamed with lipopolysaccharide (LPS) [41].

## 2. Results and Discussion

### 2.1. Preparation and Characterization of HS Extracts

HS extracts were prepared performing maceration (Mac), Ultrasound Assisted Extraction (UAE) and Microwave Assisted Extraction (MAE) exploiting ethanol/water 80/20 v/v as solvent. Mac and UAE were performed both in light and dark conditions at different temperatures. Extraction time and temperature were chosen according to literature, taking into account the stability of anthocyanins [26]. For the analytical characterization of the HS extracts, an appropriate reverse phase HPLC/UV-PAD method under gradient condition was set up. All the extracts give rise to chromatograms with a similar profile, indicating that they are characterized by a similar composition.

**Figure 1:**
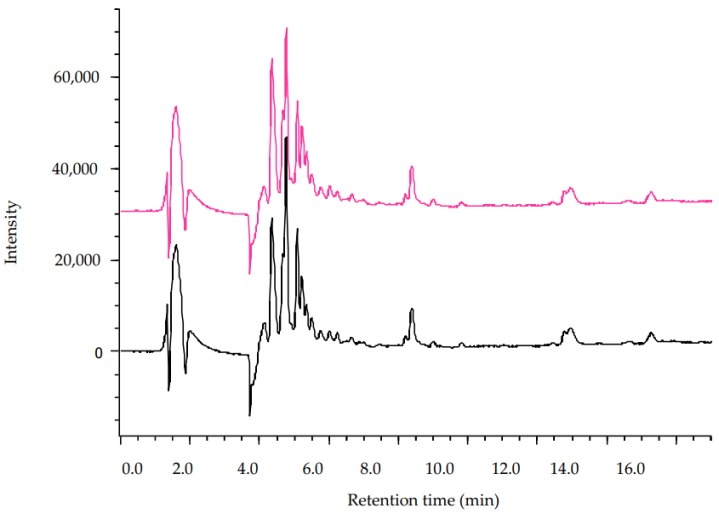
Chromatographic profiles recorded at λ = 274 nm of HS extracts obtained by Microwave Assisted Extraction (MAE, red line) and Ultrasound Assisted Extraction (UAE, black line).

In Figure 1, chromatographic profiles of MAE and UAE extracts are reported as an example.

The three extraction methodologies were then compared considering both extraction yield and free radical scavenging (FRS) activity (Table 1), evaluated by means of DPPH test, a chemical assay widely used as primary screening of antioxidant activity of natural compounds [42,43]. Overall results showed that the methodology used as well as the operating conditions (light or dark) do not affect the extraction efficiency, giving rise to extraction yields ranging from 34.6 to 40.7%. A different trend was observed for the FRS properties. Indeed, FRS% seems to be related to both extraction temperature and light or dark status, rather than the methodology applied: Mac, UAE and MAE at 45 °C under dark conditions give rise to extracts with FRS% values very close to 60%.

The results obtained highlighted that MAE is the most convenient extraction method, allowing to obtain an extract with yield and FRS activity % comparable with Mac and UAE at 45 °C under dark conditions, but in very shorter times. For this reason, such an extract was chosen for the continuation of the work.

### 2.2. Rheological Properties

In a previous work of ours [4], in situ gelling formulations, intended for the treatment of oral mucositis and esophagitis induced by cancer therapies, were developed. In particular, the formulations consisted of κ-CG, a hydrophilic sulfate marine polymer with wound healing properties and able to gelify in presence of saliva ions, hydroxypropyl cellulose (HPC) as mucoadhesive agent and CaCl_2_ as salt able to enhance the interaction κ-CG/saliva ions.

In the present work, the formulations characterized by the best rheological and mucoadhesive performances, containing κ-CG 0.4 or 0.6% w/w, HPC 1% w/w and CaCl_2_ 0.04% w/w, were loaded with HS extract at 0.2% w/w.

**Figure 2:**
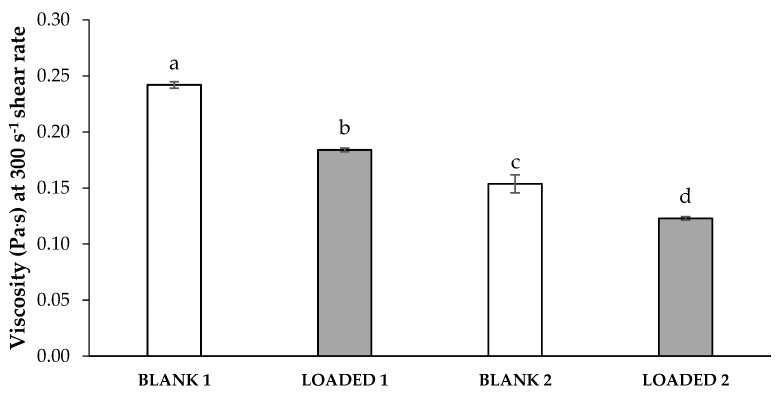
Viscosity values measured at 25 °C and 300 s^−1^ shear rate of the formulations BLANK 1 (containing 0.6% w/w κ-CG) and BLANK 2 (containing 0.4% w/w κ-CG) before and after loading of HS extract at the concentration of 0.2% w/w (LOADED 1 and 2) (mean values ± s.d.; n = 3). Anova one-way, Multiple Range Test (p < 0.05): a *vs* b–d; b *vs* c, d; c *vs* d.

Figure 2 compares the viscosity values measured at 25 °C and 300 s^−1^ shear rate of the formulations BLANK 1 (containing κ-CG 0.6% w/w) and BLANK 2 (containing κ-CG 0.4% w/w), before and after HS loading (LOADED 1 and LOADED 2). Such results represent the mean values obtained from three aliquots collected in different regions of each formulation. The low variability of the data proves the homogeneity of the samples under investigation. The same rheological measurements were performed 1h, 24h and 1 week after sample preparation and no significant viscosity variation was pointed out (data not shown). In the case of the loaded formulations, such results indicate their physical stability in the time period considered.

The presence of HS is responsible for a decrease of the formulation viscosity (Figure 2). This decrease is functional to an easy administration: a low viscosity is accompanied by a better distribution of the formulation on the application mucosa. In addition, the presence of HS is responsible for a greater capability of both the formulations to interact with saliva ions, as indicated by the normalized rheological synergism parameter (ΔSYN) calculated for LOADED 1 and LOADED 2, that was equal, respectively, to 1.44 ± 0.09 and 1.19 ± 0.06 (mean values ± s.d.; n = 3). ΔSYN values higher than 1 indicate a greater capability of the formulations to interact with saliva ions with respect to the relevant blank vehicles. Between the two loaded formulations, LOADED 1 is characterized by the highest ΔSYN value; its performance in terms of increase in viscosity when in contact with saliva ions is about one and a half times the gelling capability of BLANK 1.

**Figure 3:**
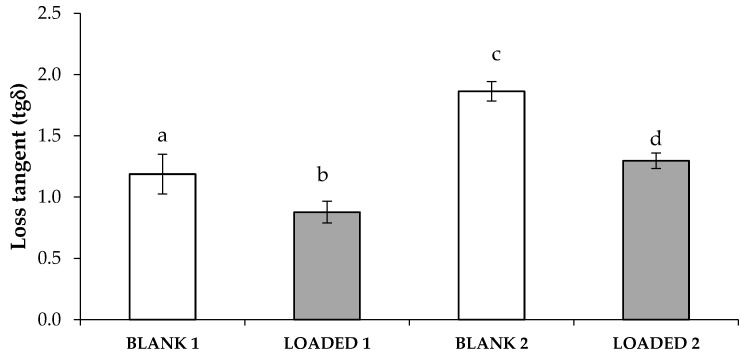
Loss tangent (tgδ) values of blank and loaded formulations upon dilution 3:1 w/w in artificial saliva (mean value ± s.d.; n = 3). Anova one-way, Multiple Range Test (p < 0.05): a *vs* b, c; b *vs* c, d; c *vs* d.

In Figure 3, loss tangent (tgδ) values of HS-loaded and blank formulations upon contact with artificial saliva are compared. It can be observed that both the loaded formulations are characterized by lower loss tangent values with respect to the relevant blanks. Since the loss tangent is calculated as the ratio between the loss (G’’) and the storage (G’) moduli that represent, respectively, the viscous and the elastic components, a lowering of tgδ indicates that the presence of HS produces a strengthening of the formulation elasticity. Moreover, the LOADED 1 formulation is characterized by a loss tangent value lower than 1, indicating that the elastic behavior is higher than the viscous one. This means that the interaction with saliva ions produces a polymeric network that, when subjected to a mechanical stress, mainly responds deforming itself and recovering the strain undergone when the stress is removed. As stated in a previous work [44], this behavior functions as a protective action of the solution towards the application site.

### 2.3. Mucoadhesion Properties

**Figure 4:**
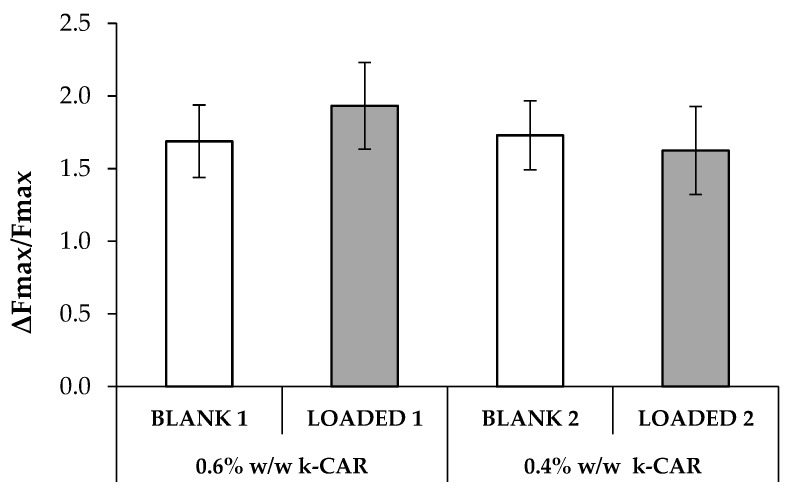
Comparison of the values of the normalized mucoadhesion parameter (ΔFmax/Fmax), observed for the blank and loaded formulations (mean value ± s.d.; n = 6). Anova one way: no significant differences.

As reported in the Part I of this research work [4], the presence of HPC at 1% w/w into the formulations is responsible for their mucoadhesive properties. To compare the mucoadhesion behavior of the blank and HS-loaded formulations on homogeneous basis, the normalized mucoadhesion interaction parameter (ΔFmax/Fmax) was calculated by normalizing the difference between the maximum detachment force in the presence and absence of mucin for the maximum force in the absence of biological substrate [36,37]. In Figure 4, ΔFmax/Fmax values of all the formulations are reported. It can be observed that the presence of HS does not significantly affect the mucoadhesive properties of the formulation.

On the basis of the results obtained, LOADED 1 formulation was chosen for the continuation of the work because it was characterized by the greatest capability to interact with the saliva ions, forming a gel with marked elastic properties and able to adhere to the mucosa.

### 2.4. In Vitro Functional Properties of LOADED 1 Formulation

**Figure 5:**
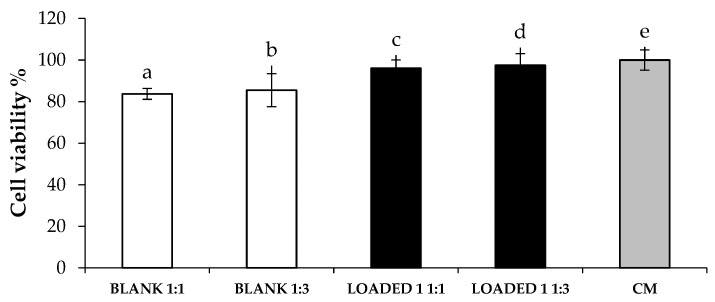
Viability % values calculated after cell contact with BLANK 1 and LOADED 1 formulations for 24 h. Two different dilutions, 1:1 and 1:3 v/v, in CM were considered. CM was used as reference (mean values ± s.d.; n = 3). Anova one-way, Multiple Range Test (p < 0.05): a *vs* b–e; b *vs* c–e.

The cytotoxic effect of both BLANK 1 and LOADED 1 formulations was investigated on human dermal fibroblasts. Figure 5 shows the percentage of living cells after treatment with the samples, upon dilution 1: 1 and 1: 3 v/v in complete culture medium (CM). All the samples tested are characterized by viability % values higher than 80%. Such a result highlights that the polymers used for the preparation of the in situ gelling formulations considered in the study are biocompatible [45]. These results demonstrate that the addition of HS extract improves the formulation compatibility with the cell substrate. In particular, LOADED 1, regardless of the dilution considered, shows viability % values that are comparable to CM and statistically higher than those obtained for BLANK 1.

In order to verify if the LOADED 1 formulation was not only biocompatible, but also able to promote cell growth, a proliferation test was performed on both blank and HS-loaded formulations. In such a test, all the samples were diluted in medium without serum (M w/s). Since serum is the medium component principally involved in promoting cell proliferation, the use of M w/s should allow to investigate the actual sample capability to enhance cell growth. Fibroblasts were seeded in the medium without serum (M w/s) and, simultaneously, put in contact with the samples in order to verify if their presence could interfere on cell growth. An aqueous solution of HS, at the same concentration used for the preparation of LOADED 1 formulation (0.2% w/w), was investigated for a better understanding of the proliferative effect of HS extract.

**Figure 6:**
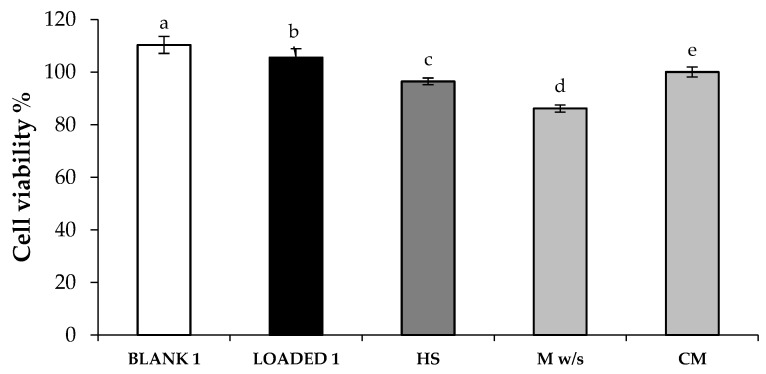
Proliferation % values calculated after cell contact with BLANK 1 and LOADED 1 formulations (diluted 1:1 v/v in M w/s) for 24 h. A solution of HS extract prepared in distilled water at the concentration of 0.2% w/w (diluted 1:1 v/v in M w/s) was also investigated (HS). M w/s and CM were used as references (mean values ± s.d.; n = 3). Anova one-way, Multiple Range Test (p < 0.05): a *vs* b–e; b *vs* c–e; c *vs* d–e; d *vs* e.

Figure 6 highlights a significant difference between cell proliferation % values of CM and M w/s, indicating the discriminating power of the test. Both blank and HS-loaded formulations, when diluted 1:1 v/v in M w/s, show viability percentages statistically higher than those obtained for the references, CM and M w/s, proving their capability to promote cell proliferation. Moreover, LOADED 1 formulation is characterized by a cell proliferation % value significantly higher than that obtained for HS, suggesting that the polymers used to prepare the formulations are able to improve extract capability to promote cell proliferation.

The capability of BLANK 1 and LOADED 1 formulations to protect cells against the oxidative damage was assessed. In particular, the antioxidant properties of HS extract, as proven above by DPPH assay, were investigated on fibroblasts treated with H_2_O_2_: both HS aqueous solution (0.2% w/w) and HS-loaded formulation were considered.

**Figure 7:**
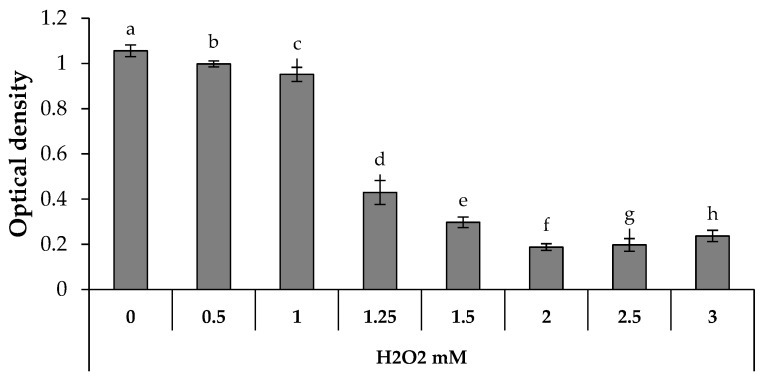
Optical density values measured after cell treatment with increasing H_2_O_2_ solutions for 24 h (mean values ± s.e.; n = 6). Anova one-way, Multiple Range Test (p < 0.05): a *vs* c-h; b *vs* d–h; c *vs* d–h; d *vs* e–h; e *vs* f–g; f *vs* h.

**Figure 8:**
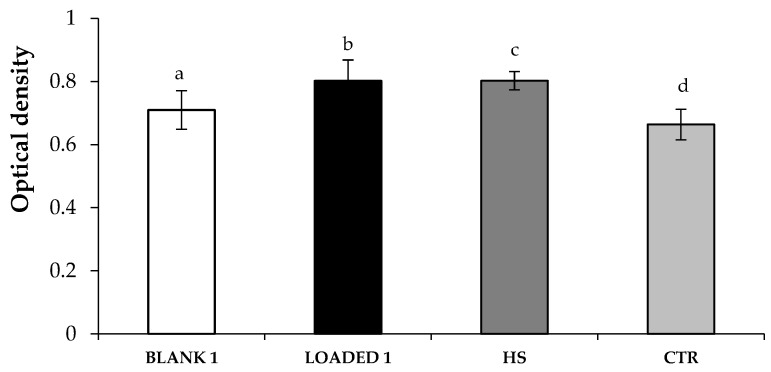
Optical density values measured after cell treatment with the samples (diluted 1:1 v/v in CM) for 24 h and, then, with H_2_O_2_ (1.25 mM) for other 24 h. Cells subjected to the oxidative stress in absence of the samples were considered as control (CTR) (mean values ± s.e.; n = 3). Anova one-way, Multiple Range Test (p < 0.05): b *vs* d; c *vs* d.

Figure 7 shows the optical density values measured after cell contact with increasing H_2_O_2_ concentrations. It can be observed that H_2_O_2_ concentrations higher than 1.25 mM are responsible for a statistically significant decrease of the optical density, when compared to the untreated cells (H_2_O_2_ concentration equal to 0). In an attempt to evaluate the antioxidant properties of the samples, 1.25 mM was selected as the H_2_O_2_ concentration necessary to induce a proper oxidative damage, without a complete cell death. Therefore, fibroblasts were treated with the samples for 24 h and, then, subjected to an oxidative stress with H_2_O_2_ at the concentration of 1.25 mM. Figure 8 points out that LOADED 1 formulation and HS solution exert an antioxidant effect, since they are characterized by optical density values higher than those observed for the cells subjected to H_2_O_2_ in absence of the samples (CTR). No statistical difference is observed between the values of LOADED 1 and HS, indicating that the vehicle does not disturb the anti-oxidant properties of HS.

As expected, BLANK 1 formulation is not characterized by an antioxidant effect, showing an optical density value similar to that measured for the CTR.

Such results can be explained considering the composition of the aqueous extract of *Hibiscus sabdariffa*, which is rich in anthocyanins and other phenolic compounds, such as chlorogenic acids. Beltràn-Debón and colleagues (2010) demonstrated that the use of HS aqueous extract preserved peripheral blood mononuclear cells (PBMCs) from the cellular death induced by H_2_O_2_. In particular, a dose-dependent resistance to the oxidative damage was observed after PBMCs co-incubation with HS and H_2_O_2_, confirming the presence of antioxidant compounds in the HS extract. The same effect was observed when PBMCs were pre-treated with HS and, subsequently, with H_2_O_2_ [46].

**Figure 9:**
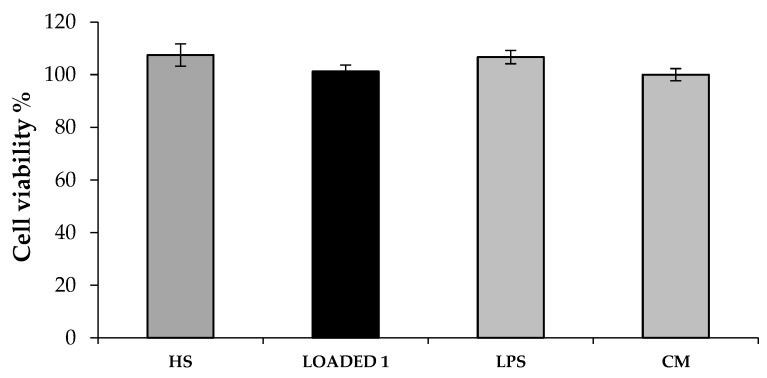
Viability % values calculated after cell treatment with the samples (diluted 1:1 v/v in CM) for 24 h and, then, with LPS solution (10 μg/mL) for other 24 h. Cells subjected to LPS-inflammation in absence of samples were considered as the negative control (LPS). CM, not inflamed cells, was used as reference (mean values ± s.d.; n = 3).

Finally, the anti-inflammatory effect of HS extract and LOADED 1 formulation was investigated on cells inflamed with lipopolysaccharide (LPS). Figure 9 reports cell viability % of the inflamed cells untreated (LPS) and treated with the formulations (BLANK and LOADED 1). It can be observed that cell viability is not affected neither by the LPS-treatment nor by the samples: no statistically significant differences are observed between the viability % values of inflamed cells in presence of the samples (HS and LOADED 1), inflamed cells in absence of the samples (LPS) and not inflamed cells (CM) (Figure 9). This result is in line with what reported in the literature [41].

**Figure 10:**
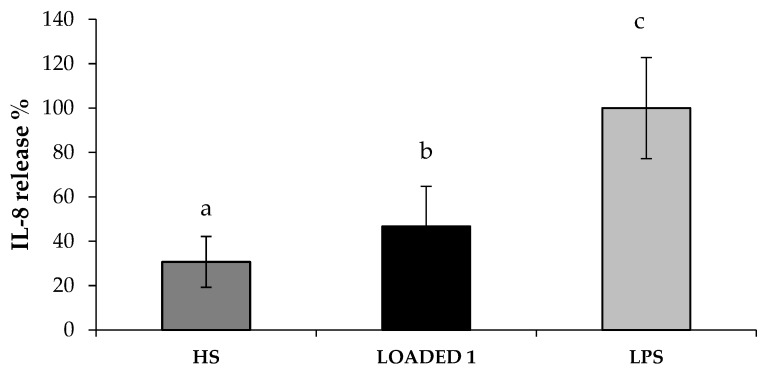
IL-8 release % values calculated after cell treatment with the samples (diluted 1:1 v/v in CM) for 24 h and, then, with LPS solution (10 μg/mL) for other 24 h. Cells subjected to LPS-inflammation in absence of samples were considered as negative control (LPS) and corresponds to 100% (mean values ± s.e.; n = 3). Anova one-way, Multiple Range Test (p < 0.05): a *vs* c; b *vs* c.

In Figure 10, IL-8 release % values observed in presence of HS extract and LOADED 1 formulation are reported. When fibroblasts were pre-treated for 24 h with LOADED 1 formulation, the LPS-induced inflammation leads to a lower IL-8 release. In particular, the percentage of IL-8 released after treatment with both HS solution and LOADED 1 formulation is statistically lower than 100% (CTR, inflamed cells in absence of samples). This indicates that HS and LOADED 1 are characterized by anti-inflammatory properties. No statistical difference is observed between the values of LOADED 1 and HS, indicating that the vehicle does not disturb the anti-inflammatory properties of HS.

## 3. Materials and Methods

### 3.1. Materials

The solvents for the extraction and the high performance liquid chromatography (HPLC grade) were supplied by Carlo Erba (Milan, Italy). Formic acid and 2,2-diphenyl-1-picrylhydrazyl radical (DPPH) were purchased from Sigma Aldrich (Milan, Italy). All solvents were evaporated under reduced pressure using a Heidolph Laborota 4000 instrument (Heidolph Instruments GmbH & Co, Schwabach, Germany).

For the preparation of all the formulations and their characterization in terms of rheological and mucoadhesive properties, the materials hereafter reported were used. κ-carrageenan (κ-CG), porcine gastric mucin type II and calcium chloride (CaCl_2_) were purchased from Sigma-Aldrich (Milan, Italy). Potassium chloride (KCl), sodium chloride (NaCl), sodium bicarbonate (NaHCO_3_) and sodium phosphate monobasic (NaH_2_PO_4_·H_2_O) were purchased from Carlo Erba Reagents (Milan, Italy). Klucel^™^ hydroxypropylcellulose (HPC) was from Ashland (Schaffhausen, Switzerland).

For experiments with Normal Human Dermal Fibroblasts (NHDF) from juvenile foreskin (PromoCell GmbH, VWR, Milan, Italy), the materials hereafter reported were used. Dimethyl sulfoxide (DMSO), Dulbecco’s Phosphate Buffer Solution (PBS), MTT (3- (4,5-dimethylthiazol-2-yl)-2,5-diphenyltetrazolium bromide), antibiotic/antimycotic solution (100×; stabilized with 10,000 units penicillin, 10 mg streptomycin, and 25 μg amphotericin B per mL), trypan blue solution, trypsin– EDTA solution and lipopolysaccharide from *Escherichia coli* O55: B5 were purchased from Sigma-Aldrich (Milan, Italy). DMEM was purchased from Corning Incorporated (Corning, NY, USA) and inactivated fetal bovine serum from Biowest (Nuaillé, France).

### 3.2. Plant Material and Extraction Procedure

Dried calyces of *Hibiscus sabdariffa* Linn were cultivated in Burkina Faso and bought in a local market. The matrix was stored in dark conditions and, at the time of use, it was cut to small size and grounded with a blade mill (A10 IKA-Werke GmbH & Co., Staufen, Germany) to obtain a homogeneous fine powder.

HS extracts were prepared by extracting 10 g of powder with 200 mL of ethanol/water 80/20 v/v for three times and comparing three different approaches: dynamic maceration, ultrasound assisted extraction (UAE) and microwave assisted extraction (MAE).

In detail, maceration and UAE (Elma Transsonic T420, Singen, Germany) were performed in four different conditions alternating light and dark, room and hot temperature (heating plate with magnetic stirring, VELP Scientifica, Milan, Italy).

On the other side, MAE was performed in a multimode microwave apparatus, using a closed-vessel system (MARSX press, CEM Corporation, Matthews, NC, USA), at 50 °C for 5 min (ramp time 1.30 min), with a power of 400 W.

### 3.3. High Performance Liquid Chromatography Analyses

High performance liquid chromatography-photodiode array (HPLC-UV/PAD) analyses were performed on a Jasco system (Cremella (LC), Italy) equipped with a Jasco AS-2055 plus autosampler, a PU-2089 plus pump and a MD-2010 plus multi-wavelength detector. Experimental data were acquired and processed by Jasco Borwin PDA and Borwin Chromatograph Software (ChromNAV 2.0 HPLC Software, Jasco-Europe, Cremella (LC), Italy).

Reverse phase chromatographic analyses were carried out at room temperature (r.t.) under gradient conditions, using a Symmetry RP-18 column (150 mm × 3.9 mm, macropore size 5 μm, mesopore size 300 Å, Waters).

The HPLC analysis conditions were initially set up to allow comparison among different HS extracts. The mobile phase was water containing 0.1% formic acid and the composition gradient was: from 10% to 75% of B in 16 min, 10% B until 3 min, followed by a re-equilibration step of 4 min; total run time of 23 min. The chromatographic run was monitored at wavelength of 245 nm.

For all analyses the flow rate was set at 1 mL/min. When necessary samples were dissolved in water (1 mg/mL) and filtered with a 0.45 μm GH Polypro (GHP) membrane before injection into the HPLC-system.

### 3.4. Free Radical Scavenging Activity

The free radical scavenging activity (FRS) of the extracts was determined by using a 2,2-diphenyl-1- picrylhydrazyl (DPPH). Briefly, dried HS extracts and a commercially available standardized green tea extract (Green Select^®^, Indena S.p.A., Milan, Italy) were dissolved in MeOH at a concentration of 2 mg/mL. 100 μL of each solution were, then, added to 3.9 mL of DPPH solution, freshly prepared by dissolving DPPH in methanol/KH_2_PO_4_ and NaOH buffer (50/50 v/v) at a concentration of 6 × 10^−5^ M, giving test solutions with final concentrations of 50 μg/mL. After 30 min of incubation at room temperature, the absorbance was measured at 515 nm by the UV-Visible.

FRS was expressed as a percent compared with the control, consisting of 3.9 mL of DPPH solution and 100 μL of methanol. The percent inhibition of the DPPH radical by the test solution was calculated using the following formula:

FRS%= [(Abs control − Abs sample)/Abs control] × 100

The analyses were carried out in triplicate.

### 3.5. Preparation of Unloaded and HS-Loaded κ-CG/HPC/CaCl_2_ Solutions

In the present work, two formulations, named as BLANK 1 and BLANK 2, containing κ-CG at 0.6 and 0.4% w/w respectively, HPC 1% w/w and CaCl_2_ 0.04% w/w, were prepared as detailed below. Briefly, κ-CG was dissolved in distilled water at 80 °C; HPC and CaCl_2_ were, then, sequentially added to each κ-CG solution and maintained under magnetic stirring at room temperature until complete dissolution of all the components occurred.

Both the formulations were loaded with dried HS extract at the concentration of 0.2% w/w and maintained under mild stirring for 3 h. Loaded formulations were, therefore, labelled as LOADED 1, when derived from BLANK 1, and as LOADED 2, when derived from BLANK 2.

Prior to each analysis, all polymeric solutions were maintained at rest for 24 h at 4 °C.

### 3.6. Preparation of Artificial Saliva

Artificial saliva was prepared dissolving KCl 1.5%, NaCl 0.43%, CaCl_2_ 0.22%, NaHCO_3_ 0.42% and NaH_2_PO_4_·H_2_O w/v in distilled water [34].

### 3.7. Viscosity Measurements

Rheological analyses were carried out by means of a rotational rheometer (MCR 102, Anton Paar, Turin, Italy) equipped with a cone plate combination (CP50-1, diameter = 50 mm; angle = 1°) as measuring system. Viscosity measurements were performed at increasing shear rates in the range 1–300 s^−1^ at 25 °C on the sample as such. In particular, three aliquots were collected in different regions of each sample and subjected to rheological characterization. Each measure was performed in triplicate. Such measurements were carried out 1 h, 24 h and 1 week after sample preparation (storage temperature: 4 °C).

In order to investigate the rheological interaction between sample and saliva ions, each formulation was diluted 3:1 w/w in artificial saliva (S) or in distilled water (W) and subjected to viscosity measurement at 37 °C.

The normalized rheological synergism parameter (ΔSYN) was calculated at 50 s^−1^, according to Equation (1):

ASYN = [(η_S_ − η_W_)/ η_W_]_LOADED_/ [(η_S_ − η_W_)/ η_W_]_BLANK_ (1)

where: η_S_ = viscosity measured at 37 °C upon dilution 3:1 w/w in artificial saliva; η_W_ = viscosity measured at 37 °C upon dilution 3:1 w/w in deionized water.

Three replicates were considered for each sample.

### 3.8. Viscoelastic Measurements

Rheological analyses were performed by means of a rotational rheometer (MCR102, Anton Paar, Turin, Italy), using a C50-1 cone (Ø = 50 mm and ϑ = 1°) as measuring system.

Sample viscoelasticity was assessed by dynamic oscillatory measurements, such as stress sweep test and oscillation test. In the stress sweep test, increasing stresses were applied at a constant frequency (0.1 Hz) and the elastic response of the sample, expressed as storage modulus G′, was measured. Such a test allows us to identify the “linear viscoelastic region”. In the oscillation test, a shear stress, chosen in the linear viscoelastic region previously determined, was applied at increasing frequencies (0.1 to 10 Hz) and G’ (storage modulus) and G’’ (loss modulus) profiles were recorded. Loss tangent value (tgδ) was calculated as G’’/G’ at 1 Hz. Measurements were performed at 37 °C on solutions upon dilution 3: 1 w/w in artificial saliva. Three replicates were considered for each sample.

### 3.9. Mucoadhesion Measurements

The mucoadhesive properties of blank and loaded formulations, after 3:1 w/w dilution in artificial saliva, were assessed at 37 °C by means of a TA.XT plus Texture Analyzer (Stable Micro Systems, Godalming, UK), equipped with 1 kg load cell and with a cylindrical movable probe (P/10C). A porcine gastric mucin dispersion (8% w/w) was prepared in artificial saliva as biological substrate.

Each sample (30 µL) was layered on a filter paper disc (Ø = 10 mm) and fixed on the movable probe. 30 µL of mucin dispersion were fixed, faced to the solution, on the sample holder. A preload of 2500 mN was applied for 300 s. The probe was then lowered to put in contact mucin dispersion and diluted sample. The probe was, then, raised at a constant speed (2.5 mm/s) up to the complete mucin-sample separation. Blank measurements were also carried out using 30 µL of artificial saliva instead of mucin dispersion. Six replicates were considered for each sample.

The maximum detachment force (Fmax, mN) was measured and the differential parameter ΔFmax was calculated according to the following Equation (2):

ΔFmax= (Fmax_mucin_ − Fmax_blank_)/Fmax_blank_ (2)

where: Fmax_mucin_ was the maximum force measured in presence of mucin and Fmax_blank_ the maximum force measured in absence of mucin (blank measurements).

### 3.10. In Vitro Studies on Fibroblast Cell Line

#### 3.10.1. Cytotoxicity Test

The cytotoxicity of BLANK 1 and LOADED 1 formulations was assessed on NHDF. Briefly, cells (100,000 cells/cm^2^) were seeded in the basolateral chamber of Transwell^®^ Permeable Supports (Corning Incorporated, Corning, NY, USA) for 24 h. The samples, diluted 1:1 and 1:3 v/v in complete culture medium (CM), were, then, placed in the apical chamber for other 24 h. CM was used as reference. An MTT assay was performed 24 h later.

Briefly, apical chambers were removed from each well and the medium in the basolateral chamber was discarded; the cells were rinsed with PBS. Subsequently, 150 μL of MTT 7.5 μM in 300 μL of DMEM without phenol red was added to each well and incubated for 3 h (37 °C and 5% CO_2_). Finally, 300 μL of DMSO, used as solubilizing agent, was added to each well, in order to promote the complete dissolution of formazan crystals, obtained from MTT dye reduction by mitochondrial dehydrogenases of living cells. The solution absorbance was measured by means of an iMark^®^ Microplate reader (Bio-Rad Laboratories Inc., Hercules, CA, USA) at 570 nm and 690 nm wavelengths after 60 s of mild shaking. Results were expressed as % cell viability by normalizing the absorbance measured after contact with each sample with that measured for CM. Three replicates were performed for each sample.

#### 3.10.2. Proliferation Test

The capability of BLANK 1 and LOADED 1 formulations to promote NHDF proliferation was assessed. Briefly, cells (50,000 cells/cm^2^) were seeded in the basolateral chamber of Transwell^®^ System (Corning Incorporated, Corning, NY, USA) and, simultaneously, the samples, diluted 1:1 v/v in medium without serum (M w/s), were placed in the apical chamber. An aqueous solution of HS extract at 0.2% w/w, diluted 1: 1 v/v in M w/s, was also investigated. M w/s and CM were used as references. An MTT assay was performed 24 h later as previously described. Three replicates were performed for each sample.

#### 3.10.3. Assessment of Antioxidant Properties

The antioxidant properties of BLANK 1 and LOADED 1 formulations and HS solution were assessed on NHDF. The concentration of H_2_O_2_ necessary to induce a proper oxidative damage, without leading to a complete cell death, was identified as follows. Cells were seeded (50,000 cells/cm^2^) overnight into a 96-well plate (Greiner Bio-One Italia S.r.l., Cassina de Pecchi, Italy) and, then, put in contact for 24 h with different H_2_O_2_ solutions, prepared in sterile distilled water with increasing concentrations (0 mM, 0.5 mM, 1 mM, 1.25 mM, 1.5 mM and 3 mM). Untreated (without samples) cells subjected to the oxidative stress were considered as the control (CTR). An MTT assay was performed 24 h later as previously described. Eight replicates were performed for each sample.

Thereafter, cells (50,000 cells/cm^2^) were treated with the samples for 24 h and, then, with H_2_O_2_ at the concentration of 1.25 mM for another 24 h. An MTT assay was performed as previously described; three replicates were performed for each sample.

#### 3.10.4. Assessment of Anti-Inflammatory Properties

The levels of IL-8 in the culture medium of NHDF (50,000 cells/cm^2^), treated with LOADED 1 formulation and HS solution for 24 h and, then, with lipopolysaccharide (LPS) at the concentration of 10 μg/mL for another 24 h, were detected by means of Human IL-8/CXCL8 ELISA kit (Sigma Aldrich, St. Louis, MO, USA) according to the manufacturer’s instructions.

Untreated (without samples) cells subjected to LPS-inflammation were considered as the negative control (LPS), while untreated cells not subjected to LPS-inflammation (CM) was used as reference. An MTT assay was also performed in order to evaluate if the inflammation could alter cell viability %; three replicates were performed for each sample.

### 3.11. Statistical Analysis

Whenever possible, experimental values of the various type of measures were subjected to statistical analysis, carried out by means of the statistical package Statgraphics 5.0 (Statistical Graphics Corporation, Rockville, MD, USA). In particular, one-way ANOVA—Multiple Range Test and Student’s t-test were used.

## 4. Conclusions

In this study, an in situ gelling formulation loaded with *Hibiscus sabdariffa* extract intended for the treatment of oral mucositis and esophagitis was developed.

Different methodologies of extraction of HS calyces were compared to evaluate the best procedure in terms of extraction yield and antioxidant activity. In this phase, a proper HPLC method was set up to compare the chromatographic profile of different extracts. The Microwave Assisted Extraction (MAE) technique was chosen because it combines a shorter extraction time and a good extraction yield, obtaining an extract characterized by good antioxidant properties.

The investigation of the formulation gelling capability by means of the rheological analysis and of the mucoadhesion properties has driven the choice of the LOADED 1 formulation as optimal. The presence of HS produces a lowering of the formulation viscosity at room temperature (behavior functional to an easy administration) and enhances κ-CG interaction with saliva ions (behavior functional to a prolonged permanence of the formulation on the action site).

The in vitro biological tests proved that the formulation was biocompatible and did not disturb the anti-oxidant and anti-inflammatory properties of the extract releasing HS substances, which were free to carry out their biological activity.

## Figures and Tables

**Table 1:** Extraction yields and antioxidant activity of the different HS extracts obtained. Antioxidant activity is expressed as a percent compared with the control.

Extraction	T (°C)	Time (min × Cycles)	Extraction Yield (g/g of Dried Calyces)	FRS %
Mac Light	r.t.	60 × 3	36.1	50.1
Mac Light	45	60 × 3	38.3	58.1
Mac Dark	r.t.	60 × 3	34.6	42.3
Mac Dark	45	60 × 3	35.2	58.9
UAE Light	r.t.	45 × 3	39.4	46.3
UAE Light	45	45 × 3	40.7	55.1
UAE Dark	r.t.	45 × 3	38.4	54.1
UAE Dark	45	45 × 3	40.1	57.5
MAE	45	5 × 3	36.3	58.9

r.t.: room temperature.
